# Interfacial Effects on the Catalytic Performance of Water-Splitting Electrocatalysts

**DOI:** 10.3390/molecules31203710

**Published:** 2026-10-16

**Authors:** Yan Wang, Jiaqi Wei, Yanyan Wu, Yuan Lu

**Affiliations:** College of Physics Science and Technology, Shenyang Normal University, Shenyang 110034, China; w1240258539@163.com (J.W.); wuyanyan_0306@163.com (Y.W.); 13390428059@163.com (Y.L.)

**Keywords:** interface engineering, electrocatalysts, hydrogen production, electronic structure modulation, water splitting

## Abstract

Electrochemical water splitting is a core component of clean and renewable energy technologies. Developing low-cost, highly active electrocatalysts is critical to enabling its large-scale deployment. Interfacial effects, which govern charge-transfer kinetics and the adsorption of reaction intermediates, play a decisive role in enhancing the activity and stability of water-splitting electrocatalysts. To clarify how interfacial effects regulate catalytic performance, this review summarizes the fundamental principles of interface engineering and recent advances in water-splitting electrocatalysis. In particular, it focuses on how interfacial structures tune the electronic structure of active sites and the adsorption behavior of intermediates through lattice strain, interfacial charge redistribution, and orbital hybridization. These mechanisms collectively enhance the performance of the hydrogen evolution reaction and oxygen evolution reaction. Finally, the review discusses the core challenges facing interface-engineered electrocatalysts for practical water electrolysis and outlines future research directions.

## 1. Introduction

The large-scale combustion of fossil fuels has caused increasing environmental pollution. The substantial emissions of carbon dioxide, sulfur oxides, and nitrogen oxides not only exacerbate global warming but also lead to more frequent occurrence of various natural disasters. To achieve sustainable development, it is necessary to develop clean and renewable fuels that can replace fossil energy. Hydrogen energy, with the advantages of high calorific value, clean combustion products, and ease of storage, is regarded as a highly promising clean energy [1]. Using renewable energy to electrolyze water for hydrogen production, such as wind power and photovoltaics, is the key to the large-scale supply of “green hydrogen” [2].

Water electrolysis consists of two half-reactions: the hydrogen evolution reaction (HER) at the cathode and the oxygen evolution reaction (OER) at the anode. Although the HER involves only a two-electron transfer, its rate is highly sensitive to the binding strength of the hydrogen intermediate (H*). The highest HER activity can be achieved at a hydrogen adsorption free energy close to zero [3]. The OER, however, involves a four-electron transfer and the sequential adsorption and desorption of multiple oxygen intermediates (OH*, O*, OOH*), resulting in slower kinetics and efficiency far below the theoretical value [4]. Noble-metal-based materials are the ideal electrocatalysts because they possess optimal intermediate adsorption energies [5]. For example, Pt, with a hydrogen adsorption free energy near zero, is the preferred catalyst for HER [6]. However, the scarcity and high cost of noble metals limit their large-scale applications in industry. Accordingly, developing high-performance alternative catalysts has become an important research direction.

Common modulation strategies include alloying, heteroatom doping and single-atom dispersion [7,8]. Pt-based alloys such as PtCo and PtNi, together with supported Pt single-atom catalysts, can maintain excellent hydrogen evolution activity [9]. Mixed Ir–Ru oxides can balance oxygen evolution activity and structural stability by adjusting the bimetallic ratio, which is a promising electrocatalysts for the oxygen evolution reaction [10]. Non-precious metal electrocatalysts based on Ni, Fe, Co, Mo and so on, have also attracted wide attention. Non-noble metal-based electrocatalysts generally suffer from low intrinsic conductivity and a limited number of effective active sites. Moreover, during long-term electrolysis, they are also prone to the dissolution, agglomeration, and structural reconstruction of their active phases [11]. To address these problems, a variety of non-precious-metal electrocatalysts have been developed [12,13]. Typical electrocatalysts include transition metal sulfides (e.g., MoS_2_), phosphides (e.g., CoP), selenides (e.g., NiSe_2_), nitrides and carbides, and hydroxides [14,15]. Taking nickel-iron catalysts as an example, by growing nickel-iron layered double hydroxide (NiFe-LDH) on carbon nanotubes (CNTs), the resulting NiFe-LDH/CNTs composite exhibits electrocatalytic activity and stability for the oxygen evolution reaction that surpass those of commercial iridium-based catalysts [16].

Interfacial effects are one of the key mechanisms responsible for the superior performance of these catalysts. Such systems contain various interfaces, including the catalyst–substrate interface, core–shell interfaces, multiphase heterojunctions, and so on [17,18,19,20]. These interfaces modulate catalytic performance through three core mechanisms. First, lattice mismatch induces local strain that shifts the d-band center and tunes the binding strength of reaction intermediates [21]. Second, the Fermi-level difference drives electron flow and interfacial charge redistribution, altering the local electron density [22]. Third, strong interfacial coupling causes orbital hybridization and forms new chemical bonds [23]. By tuning the adsorption of reaction intermediates, the interfacial effects ultimately govern the catalytic performance of the electrodes. Therefore, studying the interfacial effects and regulation mechanisms of electronic structures is significantly important for designing high-performance electrocatalysts.

To provide clear insight into how interfacial effects regulate the catalytic performance of electrocatalysts, this review systematically summarizes recent progress in enhancing performance through interface engineering. Taking core–shell, heterojunction, and catalyst–substrate interfaces as examples, it discusses how these interfaces tune the electronic structure and adsorption behavior of the active sites via lattice strain, interfacial charge redistribution, and orbital hybridization. Finally, the challenges and future prospects of interface-engineered electrocatalysts toward practical application are outlined.

## 2. Methodology

### 2.1. Research Scope

This review focuses on the interfacial regulation of water-electrolysis electrocatalysis. By reviewing experimental and theoretical studies, it clarifies the intrinsic relationships among interfacial structure, active-site electronic structure, intermediate adsorption, and catalytic performance. It covers interface-engineered electrocatalysts for the HER and OER, and discusses three modulation mechanisms: d-band electronic-structure tuning, work-function-driven interfacial charge transfer, and orbital hybridization under strong coupling. On this basis, the influence of different interfaces on activity and stability is analyzed. The cited literature emphasizes recent work reflecting current understanding and methods, while retaining a few early studies that introduced key concepts.

### 2.2. Literature Search Strategy

The literature search was organized around three topics, namely water-splitting electrocatalysts, interface engineering, and electronic regulation mechanisms. The keywords were grouped under three themes.

The keywords were categorized by theme as follows:

(1) Reaction type: including “electrochemical water splitting,” “hydrogen evolution reaction,” and “oxygen evolution reaction”;
(2) Interface structure: including “interface engineering,” “heterointerface,” “heterostructure,” “interfacial interaction,” “core–shell structure,” and “self-supported catalyst”;
(3) Regulation mechanism: including “lattice strain,” “interfacial charge transfer,” “charge redistribution,” “orbital hybridization,” and “work function.”

Relevant literature was obtained by combining similar keywords in parallel and crossing different topic groups. In addition to the database searches, the reference lists of representative studies and related reviews were screened to identify additional studies of foundational or mechanistic value.

### 2.3. Principles for Literature Selection

Studies were selected on the basis of topical relevance, representativeness, strength of evidence, and contribution to the mechanism. Priority was given to studies that define a clear interfacial construction strategy, provide structural or electronic-state information, and offer sound interpretation of catalytic behavior. Foundational early work was included alongside representative recent advances to trace how the relevant mechanistic understanding has developed. Studies with limited topical relevance, insufficient key information, or conclusions that could not be substantiated were not used as primary evidence. Studies cited for catalytic performance were required to report identifiable test conditions and corresponding performance data, with emphasis on metrics such as overpotential, Tafel slope, and stability. Those cited for mechanistic discussion were evaluated for whether they provide structural characterization, electronic-structure analysis, or computational evidence directly relevant to interfacial interactions.

### 2.4. Analytical Framework

This review is organized around an analytical framework of interface structure, the electronic structure of active sites, intermediate adsorption and reaction processes, and catalytic performance. Qualitative synthesis and descriptive comparison are used to discuss the systems reviewed. The review first discusses how interfacial effects modulate performance, and then analyzes the correlations between interface structure and HER/OER activity. For studies with interfacial features or regulatory mechanisms, their common patterns and performance differences are compared. Where multiple mechanisms operate simultaneously, their coupling effects are assessed and the contribution of each mechanism is distinguished. On this basis, by considering catalytic activity, long-term stability, and performance under practical operating conditions, key scientific issues and application limitations in interface engineering research are identified, and future research directions are discussed.

## 3. Mechanistic Modulation of Catalytic Performance by Interfacial Effects

Interfaces in electrocatalysts mainly fall into two categories: the external interface between the catalyst and the conductive substrate, and the internal interfaces within the catalyst. The interface between the active component and the support, defined as the boundary between the catalyst layer and the conductive substrate, can not only modulate the electron distribution and tune the adsorption of reaction intermediates, but also suppress particle aggregation and enhance catalyst stability [24]. According to their configuration, the interfaces can be classified into core–shell interfaces, heterointerfaces between distinct active phases, and the catalyst–substrate interface. Such interfaces disrupt the symmetry of the crystal and the periodic potential field of atoms in the interface region. Consequently, interfacial atoms generally exhibit coordinative unsaturation and bond lengths deviating from equilibrium values. The electronic structures are markedly different from those of the bulk phase. These structural differences can induce charge transfer and orbital hybridization, which in turn reshape the electronic structure of active sites and modulate the catalytic performance [25,26].

Interfacial effects on electrocatalytic activity generally originate from three mechanisms: the modulation of d-band electronic structure induced by lattice mismatch, interfacial charge transfer driven by work function differences, and orbital hybridization under strong coupling. The three mechanisms are coupled at interfaces rather than acting independently. For example, lattice strain can alter the direction and magnitude of interfacial charge transfer, while the resulting charge redistribution in turn shifts the d-band structure and alters the orbital hybridization strength [27,28]. Their combined effect is to optimize the binding of reaction intermediates—H* for the HER and OH*/O*/OOH* for the OER—and lower the energy barrier [26,29].

### 3.1. Modulation of d-Band Electronic Structure

Lattice mismatch at electrocatalyst interfaces induces an elastic strain field, which modulates d-band electronic structure. According to the d-band model proposed by Hammer and Nørskov, the adsorption strength of an adsorbate on a metal surface is mainly governed by the coupling between the adsorbate’s valence orbitals and the metal d-band [30]. The coupling creates bonding and antibonding states, and the occupancy of the antibonding states is the key factor determining the adsorption strength. Within the d-band model, a higher d-band center (closer to the Fermi level) raises the energy of the antibonding states, thereby reducing their occupancy and leading to stronger adsorption. Conversely, a lower d-band center (farther from the Fermi level) pushes the antibonding states below the Fermi level, increasing their occupancy and weakening adsorption.

**Figure 1:**
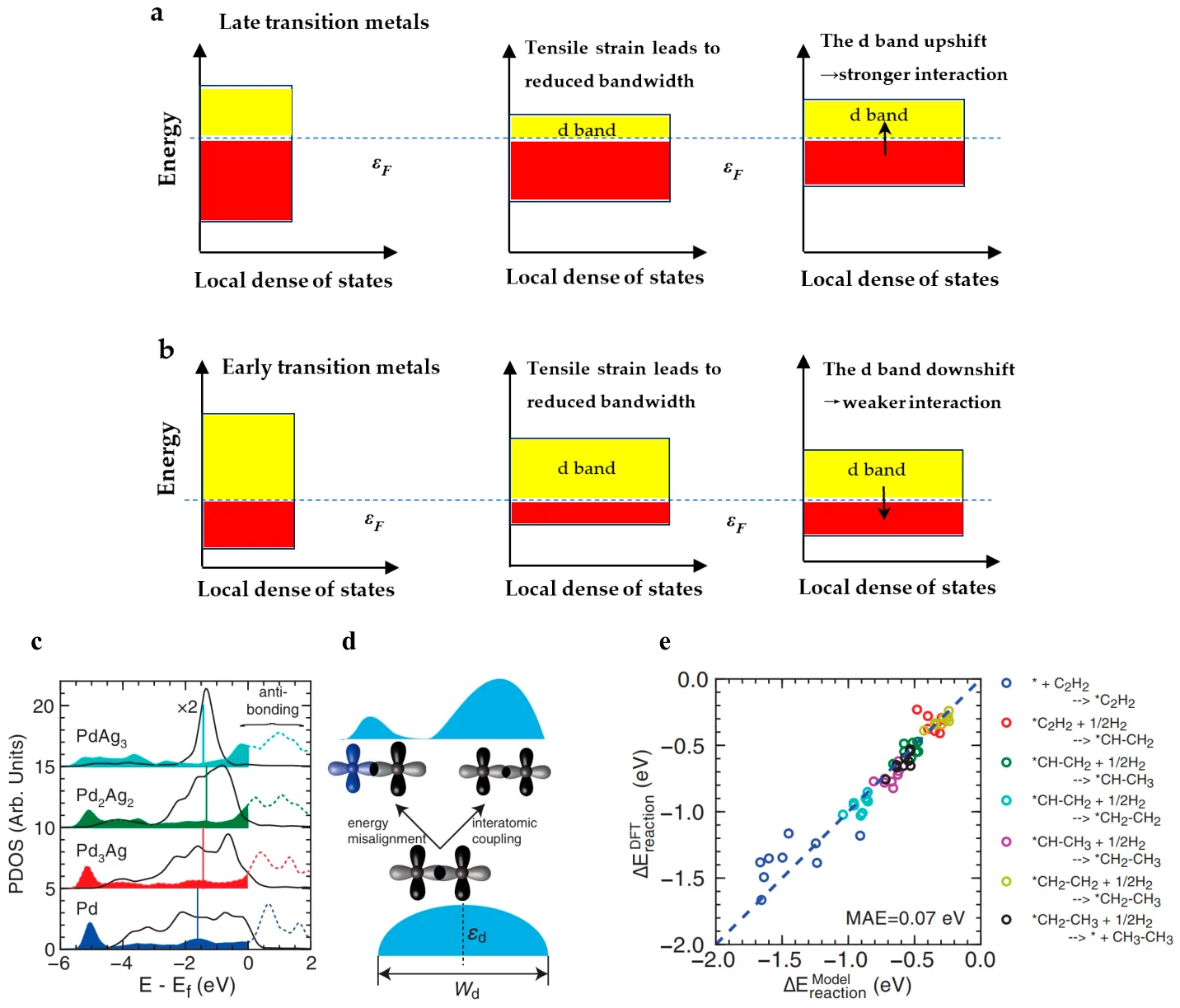
(**a**) Schematic of the influence of tensile strain on late transition metals [31]. (**b**) Schematic of the influence of tensile strain on late transition metals [31]. Copyright 2010 American Physical Society. (**c**) Schematic diagram of the d-band center variation mechanism. (**d**) Schematic illustration of the underlying mechanisms for variations in the d-band shape upon formation of alloys. [32]. (**e**) DFT reaction energies of acetylene (C_2_H_2_) hydrogenation over Pd_3_X alloys vs. model predictions, based on the ε_u_–C binding energy correlation and scaling relations for C and hydrogenated C_2_ species. [32]. Copyright 2014 American Physical Society.

Mavrikakis et al. further investigated the effect of strain on the d-band structure and adsorption energies of transition metals [28]. For late transition metals (LTMs) with more than half-filled d-bands, tensile strain reduces the overlap of d-electrons between neighboring metal atoms and narrows the d-band width. To maintain constant d-band filling, the d-band center shifts upward in energy, resulting in stronger adsorption of the adsorbate, as shown in Figure 1a. Conversely, compressive strain shifts the d-band center downward, weakening adsorption. For early transition metals (ETMs) with less than half-filled d-bands, lattice expansion results in lower adsorption energies, which is opposite to that observed in LTMs, as shown in Figure 1b [31].

Studies have shown that the local coordination geometry and chemical composition govern the d-band structure of a metal site. As shown in Figure 1c, alloying not only shifts the d-band center but also significantly modifies its width, peak shape, and electron occupancy [32]. Therefore, the d-band center alone cannot accurately describe the adsorption activity differences in complex systems such as alloys and strongly strained materials. Xin et al. proposed the upper d-band edge (ε_u_), defined as the peak position of the Hilbert transform of the d-band density of states at the active site. As illustrated in Figure 1d, ε_u_ accurately predicted C_2_H_2_ hydrogenation reaction energetics on Pd alloys. The descriptor accurately reflected antibonding state occupation and significantly improved predictions of adsorption energies and surface reaction activities.

Combining the electronic structure determined by the d-band model with machine learning (ML) regression models enables rapid prediction of adsorption energies for alloy surfaces. Taking bimetallic systems as an example, fine-tuned ML regression models combined with 14 features, including the electronic structure determined by the d-band center, can efficiently predict the adsorption energies of C, H, and O atoms across 426 fcc (111) transition metal surfaces (26 monometallic and 400 bimetallic surfaces) [33]. This interpretable ML–DFT (density functional theory) approach can be applied to transition metals and their derivatives for atomic adsorption energy prediction. For more complex binary and ternary alloys, using both the first and second moments of the d-band DOS as physics-informed features reduces the mean absolute error (MAE) of predicted adsorption energies for O, N, CH, and other adsorbates to as low as 0.13 eV [34].

In addition, high-entropy materials benefit from synergistic effects that originate from multiple principal elements; these effects manifest as lattice distortion and charge redistribution, which in turn modulate the metal d-band center relative to the Fermi level [35,36]. These effects enable quasi-continuous regulation of the electronic structure and intermediate adsorption energy. Hooch Antink et al. synthesized high-entropy spinel (CrFeCoNiMo)_3_O_4_ nanosheets containing high-valence Cr and Mo, with an overpotential as low as 255.3 mV (@10 mA·cm^−2^) [37]. The catalyst outperformed the IrO_2_ benchmark and delivered a high current density of 1 A cm^−2^ at 1.71 V in a laboratory electrolyzer. Chandrasekaran et al. trained an XGBoost model with only 70 experimental data points to predict the optimal FeCoCrMnCu-LDH from a composition space of 10626 possibilities (experimentally verified overpotential of 270 mV and Tafel slope of 74.2 mV·dec^−1^), thereby reducing the screening workload by approximately 99.3% [38]. Moreover, a FeCoNiMoZn-OH high-entropy hydroxide grown on nickel foam by pulse electrodeposition exhibited a three-dimensional porous nanosheet structure, with an overpotential of 248 mV at 10 mA cm^−2^ and a Tafel slope of 30 mV·dec^−1^ [39].

### 3.2. Interfacial Charge Transfer Driven by Work Function Differences

The work function difference across catalyst heterointerfaces (e.g., metal–substrate, metal–oxide, and metal–hydroxide interfaces) drives electron transfer until thermodynamic equilibrium is reached. This transfer establishes a contact potential difference and induces localized charge accumulation or depletion within the atomic layers at the interface. Such charge redistribution alters the electronic structure of the active sites and thus tunes their binding strength toward reaction intermediates.

The adsorption strength of these intermediates determines the kinetics of electrochemical water splitting. The hydrogen evolution reaction is mainly governed by the adsorption and desorption kinetics of hydrogen intermediates (H*) at the interface. In contrast, the oxygen evolution reaction involves a more complex process, encompassing the adsorption, transformation, and desorption of oxygenated intermediates such as OH*, O*, and OOH*. The following sections provide a detailed discussion.

#### 3.2.1. Modulation of the Hydrogen Evolution Reaction

HER efficiency is determined by the rate-determining step of the Volmer–Heyrovsky or Volmer–Tafel pathway [6]. The activation barrier of the Volmer step directly correlates with the H* adsorption free energy (ΔG_H*_) on active sites [40]. By adjusting the work-function difference, the direction and magnitude of interfacial charge transfer can be modulated, thereby tuning the hydrogen adsorption free energy. When the support has a lower work function than the metal, electrons spontaneously transfer from the support to the metal until their Fermi levels align, producing charge redistribution at the interface (accumulation on the metal, depletion on the support). The resulting increase in electron density at the metal sites can shift the metal d-band center toward the Fermi level, which reduces the occupancy of the H–metal antibonding states and thereby strengthens the metal–hydrogen bond. A moderate increase in the H adsorption energy facilitates the Volmer step, whereas excessively strong H binding hinders the subsequent Heyrovsky/Tafel desorption steps [41]. Taking (Fe,Ni)_3_P/NiCoP catalysts as an example, only a 50.9 mV overpotential was required at a current density of 10 mA cm^−2^ in an acidic electrolyte, significantly superior to single-phase NiCoP (68.9 mV), CoP NRs (77.9 mV), and Ni_2_P NRs (88.9 mV) [42].

Under alkaline conditions, the Volmer step involves the dissociation of water (H_2_O + e^−^ → H* + OH^−^). This step has a high activation barrier because it comprises the cleavage of the O–H bond, the adsorption of the resulting H*, and the stabilization and removal of the generated OH*. Such a process can be promoted by a bifunctional chemical environment, in which a metal site adsorbs H* and an adjacent oxophilic site stabilizes OH*. Interfacial charge redistribution constructs this bifunctional environment by modifying the electronic structure of the active sites [43]. Specifically, it weakens the O–H bond of the adsorbed water and thereby facilitates its dissociation, tunes the adsorption strength of the H* intermediate, and adjusts the OH* binding strength at the oxophilic sites [44]. The synergy of these effects lowers the activation barrier of the Volmer step and thus enhances the alkaline HER kinetics. For example, metal/oxide and metal/hydroxide interfaces, such as Ni/Ni(OH)_2_ and Ni/NiO, can accelerate the Volmer step by providing oxophilic sites that promote water dissociation [45,46,47].

Wang et al. reported the in situ construction of Pt–Co alloy/amorphous CoO_x_ heterointerfaces loaded on carbon powder [48]. The CoO_x_ component provided oxophilic sites that promoted water adsorption and O–H bond cleavage, while the Pt–Co alloy could facilitate hydrogen intermediate adsorption and subsequent conversion. At the same time, the interfacial charge transfer between Pt–Co and CoO_x_ led to electron accumulation at the Co sites, forming novel bifunctional active sites. DFT calculations indicated that, compared with the isolated Pt–Co alloy, the Pt–Co/CoO_x_ interface could simultaneously reduce the energy barriers for water adsorption, dissociation, and H* adsorption/desorption processes. Therefore, the Volmer step of alkaline HER was significantly promoted. Experimentally, the Pt–Co/CoO_x_/C catalyst requires only 28 mV overpotential at a current density of 10 mA cm^−2^, with a Tafel slope of 29.3 mV dec^−1^.

Both the Heyrovsky and Tafel steps involve the desorption or recombination of adsorbed hydrogen. Interfacial charge transfer therefore exerts opposite trends on the Volmer and the desorption steps. When metal sites become electron-rich, the H* adsorption strength increases, which lowers the Volmer barrier but simultaneously raises the desorption barrier [49]. Modulating the electronic structure of active sites through interfacial charge transfer from the metal substrate is one of the effective ways to overcome this limitation [50,51]. Yao et al. constructed a submonolayer of Pt on the surface of intermetallic Pd_3_Pb nanoplate (AL-Pt/Pd_3_Pb) [52]. This design increased the utilization of surface Pt atoms. Meanwhile, charge transfer from Pd_3_Pb to Pt generated strong electrostatic interactions and lowered the reaction barrier. The AL-Pt/Pd_3_Pb catalyst achieved an overpotential of merely 13.8 mV at 10 mA cm^−2^.

**Figure 2:**
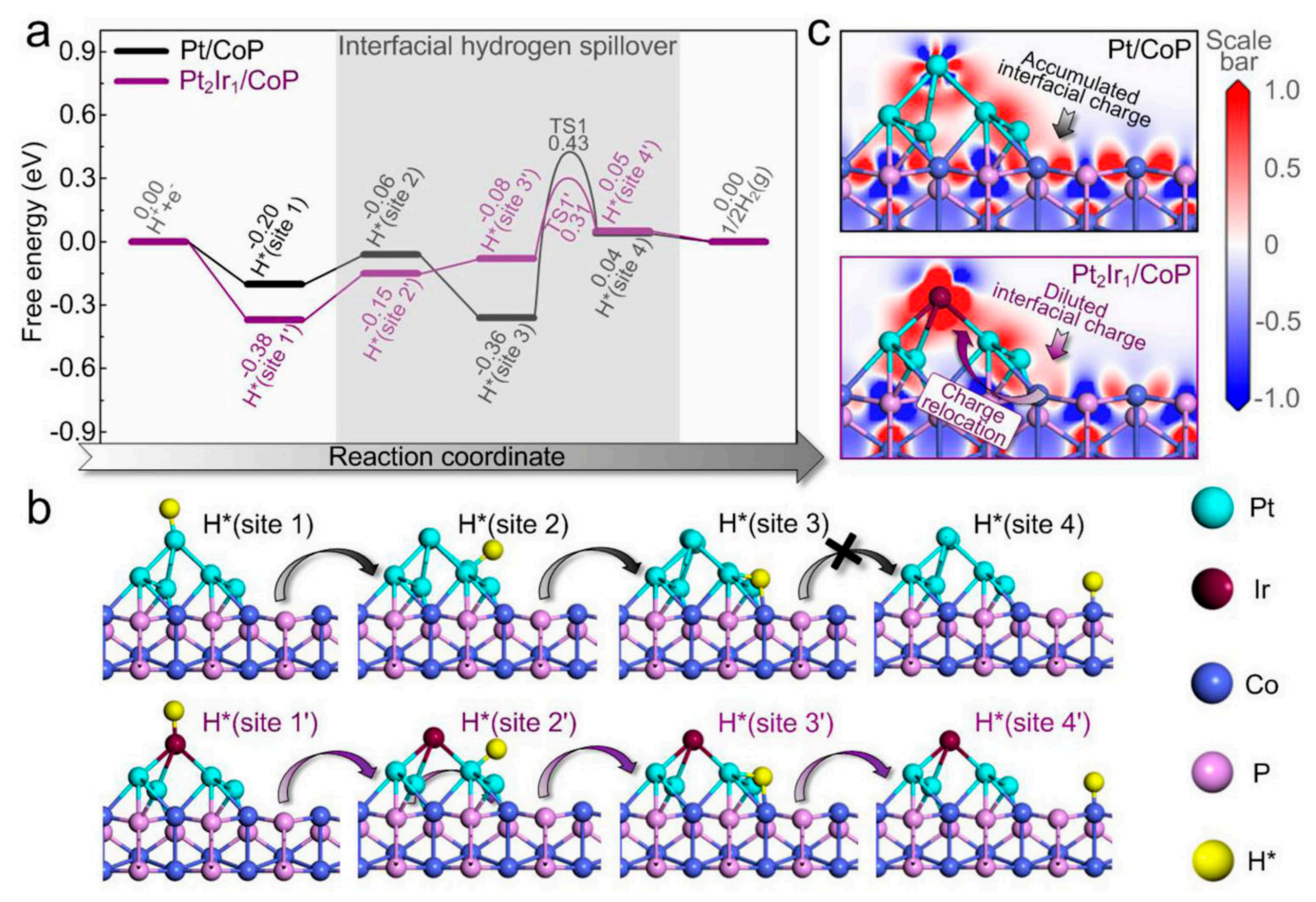
(**a**) Calculated HER free energy diagram for Pt_2_Ir_1_/CoP and Pt/CoP. (**b**) The optimized H* adsorption structures at various sites; (**c**) electron density difference map of the interfaces [54]. Copyright 2021 Springer Nature.

This adsorption–desorption trade-off makes it difficult for a single active site to optimize both processes simultaneously. To address this trade-off, one strategy is to spatially decouple the two functions. For example, H* can be adsorbed on one site and then migrate to an adjacent site for desorption or recombination, which is also called hydrogen spillover. H* is first activated and adsorbed at the metal sites (strong adsorption sites, such as Pt, Ni, etc.) via the Volmer step. H* then migrates along the surface from these strong adsorption sites to neighboring weak adsorption sites (such as oxides, carbides, or weakly binding metal surfaces). The H* at the acceptor sites rapidly desorbs and recombines into H_2_ through the Heyrovsky or Tafel steps. This process is thermodynamically unfavorable and accompanied by a high energy barrier. Two new strategies were proposed to promote hydrogen transfer at the electrocatalytic interface. On one hand, introducing a ligand environment enhances the local hydrogen coverage on the metal and reduces the barrier for interfacial hydrogen spillover. On the other hand, minimizing the work function difference (ΔΦ) between the metal and the support relieves electron accumulation and lowers the kinetic barrier for hydrogen spillover [53]. Li et al. investigated the relationship between ΔΦ and HER activity for a series of PtM/CoP catalysts [54]. DFT calculations show that the ΔΦ of Pt/CoP is approximately 0.19 eV, with a high kinetic barrier of 0.79 eV for interfacial hydrogen spillover. In contrast, the alloyed Pt_2_Ir_1_/CoP has a ΔΦ of only 0.02 eV, eliminating the interfacial electron accumulation and substantially reducing the hydrogen spillover barrier to 0.39 eV (Figure 2). The results demonstrate that a small ΔΦ induces interfacial charge redistribution, which weakens proton adsorption at the interface and promotes efficient hydrogen spillover for the HER.

#### 3.2.2. Modulation on Oxygen Evolution Reaction

The oxygen evolution reaction is a four-electron process involving multiple proton-electron coupled transfer steps and O–O bond formation [55]. It proceeds mainly through two pathways: the adsorbate evolution mechanism (AEM) and the lattice oxygen mechanism (LOM). In the AEM pathway, the oxygen-containing intermediates are generated at the metal active sites via the sequential formation of OH*, O*, and OOH*, during which the O–O bond is formed in the OOH* generation step, followed by O_2_ evolution. Weak intermediate binding may hinder water activation and OH* stabilization, whereas excessively strong binding may raise the free energy of the subsequent deprotonation, OOH* formation, or O_2_ desorption steps [6]. In addition, the linear scaling relationships among the adsorption free energies of the different intermediates restrict their independent optimization [56,57]. Consequently, enhancing OER activity requires tuning these adsorption free energies toward an optimal free-energy distribution among the elementary steps. Interfacial charge transfer provides an effective means to achieve such tuning—either by shifting the adsorption energies toward the Sabatier optimum or by selectively modifying the binding of a specific intermediate to break the scaling constraint [58]. For instance, in the CoP@Fe_3_N-Ni_4_N triphasic heterostructure electrocatalyst, the differences in work functions among CoP, Fe_3_N, and Ni_4_N induced a dual built-in electric field, driving asymmetric charge redistribution [59]. This allowed precise regulation of the local electronic structure and d-band center of the active sites, thereby optimizing the adsorption and desorption energies of intermediates during the OER process.

Wang et al. constructed a CeO_2_/Ce-Co_3_O_4_ heterojunction electrocatalyst containing both CeO_2_ nanoparticles and isolated Ce atoms [60]. The interfacial built-in electric field and the charge transfer induced by isolated Ce atoms through the Co–O–Ce configuration modulated the electronic localization of the Co sites. DFT calculations and in situ spectroscopy showed that this coexistence effect shifts the d-band center of Co sites upward, strengthens the adsorption of oxygen-containing intermediates, and increases the contribution of the lattice oxygen mechanism. The catalyst required an overpotential of only 216 mV to deliver 10 mA cm^−2^ and maintained stable operation for 100 h.

In the LOM pathway, by contrast, lattice oxygen can directly participate in O–O bond formation [61]. This process is generally described as a Mars-van Krevelen pathway [62]. Initially, oxygen ligand holes are generated in the O 2p band, rendering the lattice oxygen electrophilic. The electrophilic lattice oxygen then undergoes O–O coupling with adsorbed oxygen-containing intermediates (or neighboring lattice oxygens), forming peroxide-like species that subsequently evolve as O_2_ while leaving an oxygen vacancy. Finally, the oxygen vacancy is replenished by H_2_O or OH^−^ from the electrolyte, completing the catalytic cycle.

Interfacial charge transfer can promote the above processes by modulating the electronic structure of the active oxides. When the oxide contacts a component with a higher work function (i.e., a lower Fermi level), electrons transfer from the oxide to that component, raising the oxidation state of the active metal sites and shifting the metal d states to lower energy relative to the O 2p band. The resulting decrease in the d–p energy separation lowers the charge-transfer energy and enhances d–p hybridization (metal–oxygen covalency). This enhanced covalency shifts the O 2p-derived states closer to the Fermi level, thereby promoting the formation of ligand holes and imparting electrophilicity to the lattice oxygen; consequently, the energy barrier of the rate-determining step in the LOM pathway is reduced [63]. Using La_1−x_Sr_x_CoO_3−_δ perovskites as model systems, Grimaud et al. demonstrated by in situ ^18^O isotope-labeled mass spectrometry that enhanced metal–oxygen covalency can trigger lattice oxygen oxidation and thereby significantly improve OER activity [64].

Jia et al. constructed a CuO_x_/Co_3_O_4_ heterointerface, where the lattice oxygen characteristics near the heterointerface were effectively modulated, promoting the reaction pathway to shift from AEM to LOM [65]. In situ X-ray absorption fine structure spectroscopy further confirmed that interface engineering stabilized the valence state of Co during the OER process and mitigated Co corrosion. This catalyst exhibited excellent OER activity and overall water-splitting stability in fresh water, natural seawater, and high-salinity wastewater, with an overpotential of only 308 mV at 100 mA cm^−2^ and stable operation for 100 h at 500 mA cm^−2^. The above interface regulation strategy is universal. Oxide heterostructures such as FeOOH/Co(OH)_2_ and CeO_2_/FeOOH have been confirmed to promote lattice oxygen activation and maintain the stable operation of the LOM pathway [66,67].

### 3.3. Orbital Hybridization Under Strong Coupling

For heterogeneous interfaces or adsorption systems, the orbitals of the two components couple through wavefunction overlap. Orbitals of comparable energies hybridize into bonding and antibonding states, thereby shifting the metal d-band center and reconstructing the interfacial electronic structure [21,68]. This electronic restructuring, in turn, tunes the adsorption energies of the reaction intermediates, influencing the thermodynamics and kinetics of adsorption and desorption [69]. It also promotes interfacial charge redistribution and electron transfer, which can steer the reaction along a more favorable pathway. As a result, the catalytic activity can be enhanced. Such hybridization effects have been widely reported at interfaces such as metal–sulfide, metal–oxide, and metal–hydroxide [70,71,72].

The d–d hybridization is a common form of orbital coupling. It occurs primarily at metal–metal or metal–compound interfaces where the two metal centers possess similar d-state energies [73]. For example, Ru–Mo bonds induced strong d–d hybridization that upshifted the Mo d-band center from −1.079 to −0.929 eV, shifting ΔG_H*_ toward thermoneutral (−0.21 eV) and thereby accelerating alkaline HER kinetics [74]. For CoMoP/Cr(OH)_3_, strong interfacial coupling induced d–d hybridization that enhanced interfacial charge transfer, while the reconstructed interface exposed abundant active sites [75]. These effects contribute to the low HER/OER overpotentials of 88 and 207 mV at 10 mA·cm^−2^, respectively. Notably, d–d coupling can also drive phase transitions through the associated charge transfer. In MoS_2_@Co_3_S_4_ dual heterojunctions, charge donation from Co to Mo induces the 2H-to-1T transition of MoS_2_, and the resulting heterojunction combines the high conductivity of the 1T phase with the moderate intermediate adsorption of the 2H phase [76].

**Figure 3:**
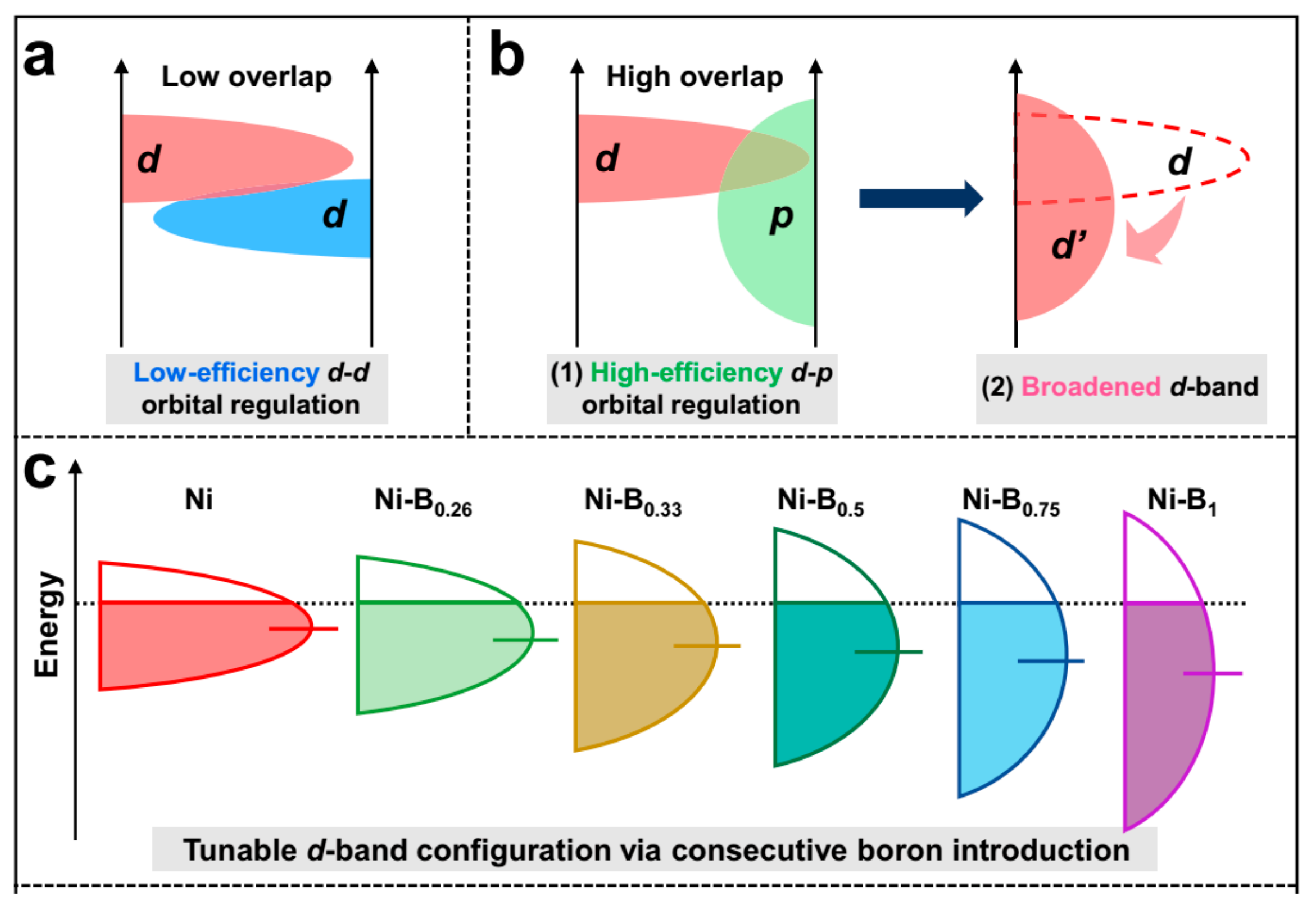

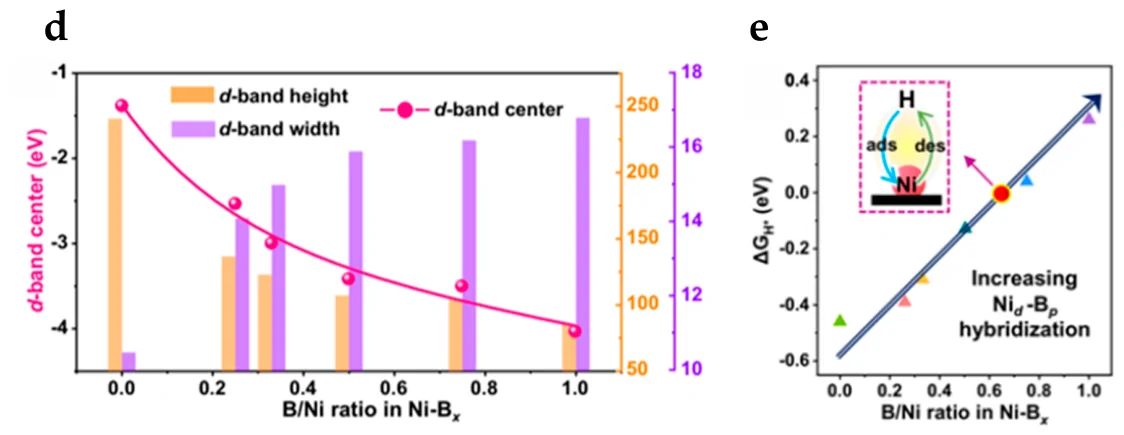
Fine-tuning of d–p hybridization in Ni–B_x_ cocatalysts. (**a**) Low-efficiency d–d orbital regulation. (**b**) Broadening of the d band via high-efficiency d–p orbital regulation. (**c**) Consecutive d-band broadening and downshift of the d-band center in composition-tunable Ni–B_x_. (**d**) Calculated d-band parameters (height, width, and center) of various Ni–B_x_. (**e**) Calculated ΔG_H*_ values of different structures [82]. Copyright 2025 Springer Nature.

The p–d hybridization is another common type of orbital coupling, in which metal d orbitals mix with the p orbitals of main-group elements. It occurs not only at metal/non-metal (or metal/support) interfaces but also within intermetallic and metal–metalloid compounds. In transition metal phosphides (e.g., CoP, Ni_2_P, MoP, FeP), metal d orbitals hybridize with P 3p orbitals, reshaping the electronic distribution of the surface active sites [77,78]. In the NiCoP/Ni_5_P_4_ heterojunction, interfacial d–p hybridization strengthened the covalent Ni/Co–P bonds and shifted the d-band center upward by 0.061 eV, which enhanced OH^−^ adsorption and facilitated water dissociation [79]. The ordered heteroatomic bonding in intermetallic compounds not only exploits such hybridization effects but also enhances thermodynamic stability, as reflected in their more negative formation enthalpies. For instance, strong d–p hybridization between Ir and Sn in carbon-supported Ir_3_Sn_7_ (Ir_3_Sn_7_/C) yielded excellent overall water-splitting performance over a wide pH range, with Ir loadings as low as 13.60% [80]. Furthermore, calculations showed that d–p hybridization between Ga and Pd modified the electronic structure of Pd and improved its adsorption affinity toward reaction intermediates [81]. Similarly, introducing B into Ni–B_x_ progressively enhanced Ni 3d–B 2p hybridization, broadening the Ni d band and tuning the d-band center (Figure 3) [82]; this modulation optimizes the H adsorption/desorption kinetics and thereby improves the hydrogen evolution activity.

## 4. Application of Interface Engineering in Electrocatalysis

In electrocatalytic water splitting, charge transfer, water adsorption and activation, the conversion of reaction intermediates, and the desorption of gaseous products all occur within the interface region. The OER suffers from intrinsically sluggish kinetics owing to its multi-electron transfer, and in alkaline media the HER is limited by the high energy barrier of the water-dissociation (Volmer) step. In addition, interfaces are prone to reconstruction, dissolution, and deactivation during long-term operation, leading to a continuous loss of catalytic activity. To address these issues, diverse interface engineering strategies have been developed, including regulating the electronic states of the active sites and the adsorption of intermediates through interfacial charge redistribution, optimizing active-site configurations via coordination-environment remodeling, and promoting charge transfer and redistribution through built-in electric fields. The following section focuses on three representative interface configurations: core–shell structures, transition-metal-based heterostructures, and substrate–catalyst interfaces. The interfacial mechanisms and recent advances of each are discussed in the following subsections.

### 4.1. Core–Shell Interface

The lattice strain induced by the lattice mismatch in core–shell structures provides a convenient means to regulate the electronic structure of surface-active sites. Such interfacial effects are confined to a limited number of atomic layers adjacent to the core. Consequently, the coupling between the surface-active sites and the interface weakens as the shell thickness increases. As a result, the activity is typically maximized at an intermediate shell thickness that balances strain retention against stability and site density. For example, the shell thickness of Pd@Pt core–shell nanoparticles was precisely tuned from 0.4 to 1.2 nm. The oxygen reduction reaction activity did not increase monotonically with thickness; instead, it reached a maximum at ~0.94 nm (several atomic layers), where high stability was also retained [83].

Similarly, the Pd_3_Pb@Pt system achieves optimal HER performance at a shell thickness of about three atomic layers [84]. The magnitude of the interfacial strain also influences the activity in a nonlinear manner. He et al. showed that the lattice strain in Pt(100) shells can be varied continuously from −5.1% to +5.9% by exploiting the reversible swelling and contraction of Pd-based nanocubes during phosphorization and dephosphorization, yielding a volcano-shaped dependence of HER activity on strain [85].

#### 4.1.1. Hydrogen Evolution Reaction

Pt-based core–shell electrocatalysts achieve outstanding performance with minimal precious-metal content. The Pt shell consists of only a few atomic layers, and most Pt atoms are exposed as surface active sites. Electronic coupling and lattice mismatch between the core and shell modulate the d-band center of Pt and tune the hydrogen adsorption energy. For example, a Rh-core@Pt-shell catalyst (Rh@Pt_2_L) was designed, in which DFT calculations indicate that the electronic and strain effects cause a moderate downshift of the d-band center [86]. Its overpotential at 10 mA cm^−2^ in alkaline media is only 5 mV, far superior to commercial Pt/C (44 mV) and Rh/C (42 mV). Similarly, an ultrathin Pt shell grown on an fcc/hcp heterogeneous PtNi alloy core (PtNi@Pt) requires only 12.9 mV at 10 mA cm^−2^ in 1 M KOH [87]. Tuo et al. prepared a Pt_1_Cu_3_/CB (carbon black) catalyst with a Pt–Cu core and a Pt-rich shell only 3–4 atomic layers thick. Pt surface segregation forms this ultrathin shell, which delivers a high electrochemical active surface area of 24.7 m^2^ g_Pt_^−1^ [88]. Meanwhile, electron transfer from Pt to Cu downshifts the d-band center of Pt. This weakens the binding of H* intermediates to a near-optimal level. Consequently, the catalyst reaches 10 mA cm^−2^ at only 10 mV in 0.5 M H_2_SO_4_ and 17 mV in 1.0 M KOH.

**Figure 4:**
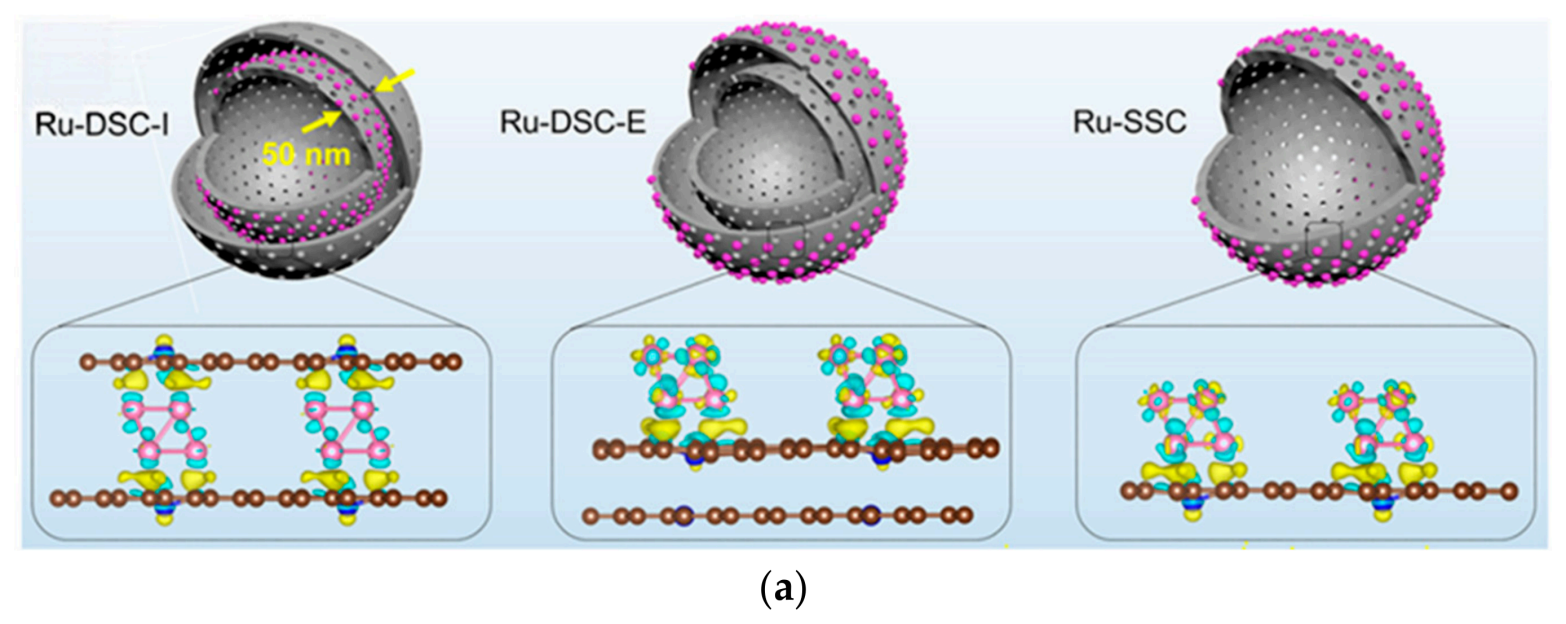

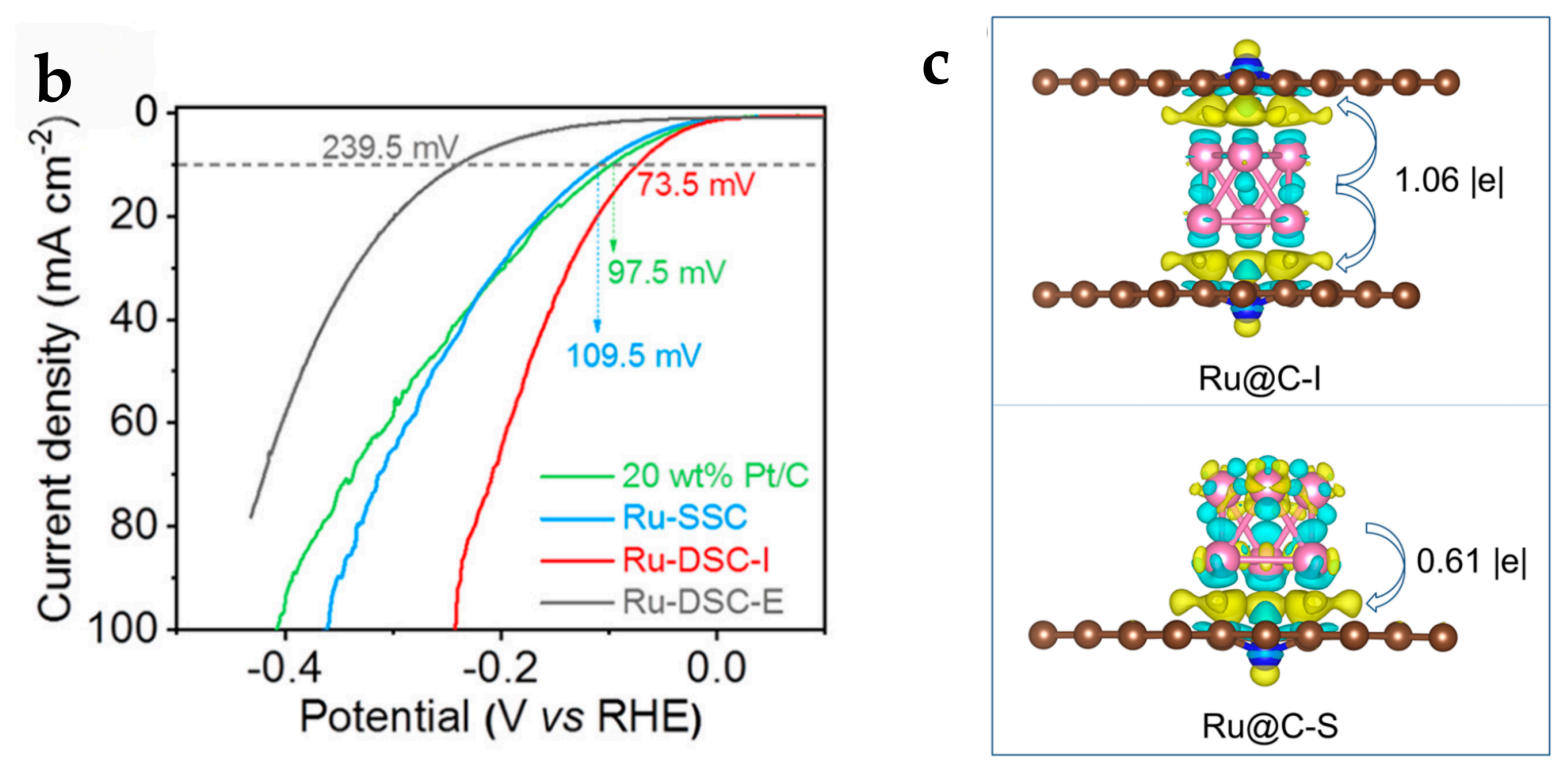
(**a**) Schematic representation of Ru-SSC, Ru-DSC-I, and Ru-DSC-E. (**b**) HER polarization curves of Ru-SSC, Ru-DSC-I, Ru-DSC-E, and commercial Pt/C. (**c**) DFT calculations of electron density difference (EDD) and Bader analysis for Ru@C-I and Ru@C-S [89]. Copyright 2024 American Chemical Society.

To address the aggregation of Ru nanoparticles and the sluggish H desorption caused by strong Ru–H bonds in alkaline HER, Liu et al. proposed a double-shell interlayer confinement strategy [89]. By altering the adsorption sequence of Ru^3+^, Ru was selectively deposited onto either the inner shell (Ru-DSC-I) or the outer shell (Ru-DSC-E) of a double-layer nitrogen-doped carbon nanoreactor, as shown in Figure 4a. In Ru-DSC-I, the confinement effect of the double shell reduced the Ru particle size to 0.9 nm, achieving an overpotential of only 73.5 mV at 10 mA cm^−2^ in 1 M KOH (Figure 4b), and stable operation at a high current density of 100 mA cm^−2^ for 24 h. The double-shell confinement created two coupled interfaces. DFT calculations indicate that the number of electrons transferred from Ru to the carbon substrate increases from 0.61 |e| (single shell) to 1.06 |e| (double shell) (Figure 4c), while the Ru d-band center downshifts from −2.54 to −3.70 eV. The free-energy changes for water dissociation and H_2_ generation are reduced to −0.24 and −0.10 eV, respectively. Compared with the single-shell nanoreactor, the double-shell configuration simultaneously achieves confined stabilization of ultrasmall Ru nanoparticles and the regulation of the electronic structure.

In alkaline HER, the electronic-structure requirements for water dissociation and for hydrogen adsorption/recombination are contradictory, making it difficult for a single site to accommodate both simultaneously. Distributing the two types of elementary steps over active sites with different chemical environments has become an important strategy to optimize alkaline HER kinetics. Subbaraman et al. demonstrated the bifunctional mechanism on Pt/Ni(OH)_2_, in which Pt provided sites for H adsorption and recombination while Ni(OH)_2_ promoted water dissociation [90]. Guided by this concept, metal/oxide (hydroxide) core–shell catalysts such as Ni/NiO and Ni/Ni(OH)_2_ have been studied systematically. Wan et al. reported a unique design of Ni(OH)_2_-clothed Pt-tetrapods [91]. An amorphous Ni(OH)_2_ shell dissociated water and acted as a proton-conductive layer that isolated the Pt core from bulk alkaline electrolyte and supplied protons to the underlying Pt sites. This created an acid-like local environment, enabling acid-like HER kinetics with the lowest Tafel slope of 27 mV dec^−1^ and record-high specific and mass activities. Silambarasan et al. showed that hydrated and dehydrated oxophilic sites on nickel hydroxide (Ni(OH)_2_) coated on a nickel (Ni) catalyst can improve HOR/HER kinetics [92]. Electrochemical measurements combined with DFT calculations indicate that, within the catalyst surface layer, water molecules that bridge adsorbed hydrogen and oxygen species can create an acid-like environment at the electrochemical interface. This is a mechanism that goes beyond the traditional bulk and interface descriptions.

Phosphide/sulfide heterostructures are also commonly used to achieve dual-site synergy. Metal cations such as Co and Ni serve as water-dissociation active centers, while anionic sites such as P and S modulate the electronic structure of the metal sites through interfacial charge transfer [93]. For example, a CoP@Ni_2_P nanoarray (CoP nanowire core @ Ni_2_P nanosheet shell) develops dense heterogeneous interfaces between the core and shell via co-phosphidation. Owing to the synergistic effect between the two phases, an overpotential of only 101 mV is required to reach 10 mA cm^−2^ in 1 M KOH [94]. Huang et al. reported core–shell Ni_2_P@CoP nanoarrays on nickel foam (Ni_2_P@CoP/NF) with a large specific surface area. These formed abundant heterostructure interfaces that can provide more active sites and enhanced conductivity, with an overpotential as low as 61 mV at 10 mA cm^−2^ [95]. Chen et al. fabricated crystalline Ni_3_S_2_ nanorods modulated by low-crystalline NiCoS_x_ (NiCoS_x_@Ni_3_S_2_) through an ion-exchange strategy [96]. At the NiCoS_x_/Ni_3_S_2_ heterointerface, the work-function-induced built-in electric field, together with Ni–S–Co bond bridges, drove electron transfer from Ni_3_S_2_ to NiCoS_x_. This shifted the d-band center, facilitating water dissociation and balancing the adsorption and desorption of H*. As a result, the optimized NiCoS_x_@Ni_3_S_2_ shows low HER/OER overpotentials of 346/520 mV at 1000 mA cm^−2^.

Table 1 summarizes representative core–shell structured hydrogen evolution reaction electrocatalysts reported between 2023 and 2026, comparing the core/shell composition, electrolyte, overpotential at 10 mA·cm^−2^, Tafel slope, and long-term stability of each system.

#### 4.1.2. Oxygen Evolution Reaction

In the oxygen evolution reaction, core–shell structures can directionally regulate the electronic structure of active sites in the shell through charge transfer, lattice strain, and ligand effects. Varying the core composition tunes the shell’s electronic structure and the adsorption energy of oxygen intermediates, thereby modulating the adsorption energy and intrinsic OER activity [107]. For example, when the core phase of the PtNi core–shell catalyst was changed from metallic Ni to Ni_4_N, the Pt d-band center was up-shifted [108]. The specific activity and mass activity reached approximately 4.5 and 6.5 times those of commercial Pt/C, respectively. Meanwhile, the Ni_4_N core also promoted the diffusion of inner-layer Pt to surface defect sites, significantly improving cycling stability.

In addition, the interfacial coupling between a one-dimensional oxide core and a layered double hydroxide shell can significantly improve the intrinsic kinetics of active sites. A hierarchical CuO@NiCo-LDH core–shell nanoarray on copper foil (CuO@NiCo LDH/CF) achieved a geometric current density of 20 mA cm^−2^ at a low overpotential of 256 mV in 1.0 M KOH [109]. Benefiting from the more continuous electron-transport pathways provided by the oxide core relative to the LDH shell, transition-metal-oxide@LDH core–shell catalysts delivered excellent activity and stability for the OER in alkaline electrolytes. A 3D open-structured CoO@NiFe LDH/NF catalyst was prepared via dielectric barrier discharge microplasma. It delivered an overpotential of only 225 mV at 20 mA cm^−2^ for the OER in alkaline media and excellent long-term stability (>80 h) [110]. Combining a metallic alloy core with an amorphous hydroxide shell offered significant advantages at large current densities. The NiFe/NiFe–OH core–shell electrode delivered an OER overpotential as low as 296 mV at 500 mA cm^−2^ in 1 M KOH [111]. Moreover, a two-electrode electrolyzer with NiCo/NiCo–OH (cathode) and NiFe/NiFe–OH (anode) operated stably at 1000 mA cm^−2^ for more than 300 h without obvious decay. As another type of interfacial structure, a hierarchical FeOOH@Ni_3_N heterostructure provided fast electronic pathways and efficient gas transport, maintaining 500 and 1000 mA cm^−2^ with overpotentials of only 284 and 311 mV [112]. Li et al. prepared a crystalline/amorphous NiO_x_ microbelt superstructure, comprising crystalline NiO cores and amorphous Ni^3+^/Ni^2+^ shells [113]. The core–shell interface, rich in vacancies and defects, offered abundant active sites. Meanwhile, electrochemical activation drove in situ reconstruction, which thickens the amorphous shell and optimizes the OER-active phase. The synergistic interaction resulted in high OER activity and long-term stability.

Under the highly oxidative potentials and strongly alkaline or strongly acidic conditions of the OER, metal dissolution, surface over-oxidation, phase separation, and particle aggregation are the primary causes of catalyst deactivation. A stable shell can serve as a physical barrier that isolates the core phase and its soluble components from the electrolyte, thereby suppressing their dissolution and outward diffusion. It also localizes over-oxidation and phase separation within the shell, preventing these processes from propagating into the core. As a result, the core–shell architecture is preserved during prolonged operation, sustaining interfacial charge transfer and a stable active surface. The FeO_x_ shell, in which corner-sharing octahedra cross-link adjacent layers, enhances structural rigidity and restricts the diffusion of OH^−^ and H_2_O [114]. It prevents the rapid oxidation of the amorphous NiP_x_ core into Ni_3_(PO_4_)_2_ and Ni(OH)_2_. For Ni_x_S_y_@MnO_x_H_y_, the MnO_x_H_y_ overlayer effectively suppresses electrochemical corrosion under high current densities, markedly improving the stability [115].

Table 2 summarizes representative core–shell structured oxygen evolution reaction electrocatalysts reported between 2023 and 2026, comparing the core/shell composition, electrolyte, overpotential at 10 mA·cm^−2^, Tafel slope, and long-term stability of each system.

### 4.2. Transition-Metal-Based Heterostructures

Owing to their tunable compositions, abundant interfacial structures, and the synergistic electronic modulation arising at the interfaces, together with relatively low preparation costs compared with noble-metal (e.g., Pt, Ir, Ru) catalysts, transition metal-based heterostructures have attracted increasing attention in water electrolysis. This section reviews recent research progress on several representative transition metal-based heterostructure systems, including transition metal–oxide, transition metal–phosphide, and transition metal–sulfide heterostructures.

#### 4.2.1. Metal–Oxide Heterointerfaces

The heterogeneous interface formed between the metal and the oxide is a classic system that has been extensively studied. Owing to the work function difference between the two phases, electrons transfer spontaneously until the Fermi levels align, redistributing the electron density of the metal sites and thereby tuning their d-band centers. In alkaline HER, the highly conductive metallic phase provides a continuous electron-transport pathway, while oxygen vacancies and coordinatively unsaturated sites on the oxide side facilitate H_2_O dissociation (Volmer step) [128]. Gong et al. prepared a NiO/Ni heterostructure on oxidized carbon nanotubes. Owing to the nanoscale NiO/Ni interface, the resulting heterostructure exhibited HER activity comparable to that of Pt/C [129]. Liu et al. synthesized abundant intimately contacted MoO_2_–Ni heterostructures [130]. Experimental and theoretical analyses confirmed that charge accumulates at the MoO_2_–Ni interfaces, where introducing Ni shifted the dominant active centers from Mo to interfacial O. The introduction of Ni elevates the O 2p band of MoO_2_, which strengthens H* adsorption, shifting it from too weak toward a more optimal strength, and accelerates the HER kinetics.

For the OER, the oxide phase (often in situ reconstructed into (Oxy)hydroxides) usually serves as the active phase [131]. Heterointerfacial coupling with the metallic phase improves the electronic conductivity of oxides by interfacial charge transfer, promotes the formation of high-valent metal active sites and optimizes their adsorption energies, thereby lowering the energy barrier of the rate-determining step. Wang et al. developed a Ru/RuO_2_–Co_3_O_4_ catalyst that couples a RuO_2_/Co_3_O_4_ heterointerface with vacancy engineering [132]. Measurements revealed that interfacial charge transfer from Co_3_O_4_ to RuO_2_ lowered the Ru valence state and weakened Ru–O covalency, while the oxygen vacancies enhanced intrinsic activity. The catalyst delivered a 226 mV overpotential at 10 mA cm^−2^ and retained high stability over 19 h in 0.1 M HClO_4_.

**Figure 5:**
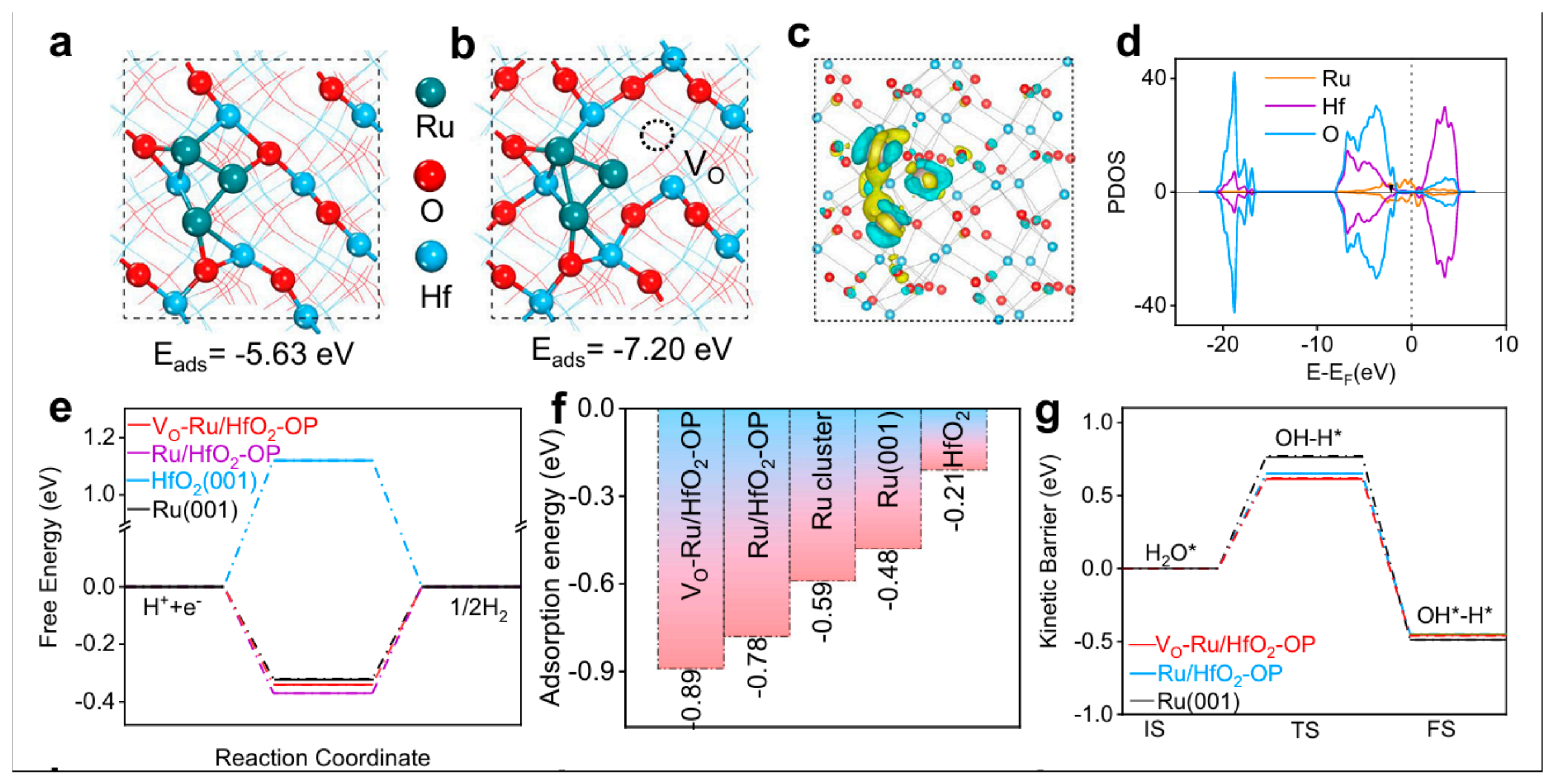
(**a**,**b**) adsorption energies of Ru_3_ clusters loaded on HfO_2_-OP and V_o_-HfO_2_-OP. (**c**) Differential charge density for the Ru_3_/V_o_-HfO_2_ interface (isovalue = 0.001 e Å^−3^); yellow and olive refer to charge gain and loss; (**d**) PDOS plots of Ru, Hf and O in V_o_-Ru/HfO_2_-OP; (**e**) HER free energy profiles relative to the standard hydrogen electrode, together with (**f**) the water adsorption energies; (**g**) the water dissociation kinetic barriers calculated at the active sites of the various catalysts. IS, TS and FS stand for the initial, transition and final states, respectively [134]. Copyright 2019 The Royal Society of Chemistry.

Moreover, the bonding interaction at the metal–oxide interface can effectively anchor active sites and inhibit the migration and aggregation of metal particles during the reaction process. By tuning the (111)/(100) facet ratio of the Co_3_O_4_ support, a facet-junction Ru/Co_3_O_4_ heterointerface was engineered [133]. This architecture substantially amplified the metal–support interaction and triggered a redistribution of interfacial charge, thereby increasing the HER activity by 560% relative to the conventional counterpart. Li et al. further tuned the density of Hf–O–Ru bonds and oxygen vacancy concentration in HfO_2_ supports to strengthen the metal–support interaction, which improved the anchoring and dispersion of Ru and thus achieved a mass activity of Ru/HfO_2_ about 20 times that of commercial Pt/C (as shown in Figure 5), with an overpotential as low as 39 mV (at 10 mA cm^−2^ in 1 M KOH) [134]. Lai et al. utilized oxygen vacancies as electronic bridges to strengthen the metal–support interaction between Ru nanoparticles and TiO_2_-x supports. This approach enhanced the anchoring of Ru nanoparticles on the support and effectively prevented the detachment and agglomeration of active species during catalysis. As a result, the overpotential for acidic HER was as low as 11 mV, with stable operation maintained for 350 h [135].

#### 4.2.2. TMD-Based Heterostructures

Transition metal dichalcogenides (TMDs) possess a graphite-like layered structure with high specific surface area and abundant edge configurations [136]. In 2007, Jaramillo et al. experimentally demonstrated that the HER activity of MoS_2_ scales linearly with the number of edge sites, by tuning the size of MoS_2_ nanoparticles and quantitatively enumerating edge sites via scanning tunneling microscopy [137]. This finding established the classical understanding that edge sites, rather than basal plane sites, are responsible for HER activity, although later studies have shown that basal planes can also be activated by defects, doping, or strain. In addition, most 2H-phase TMDs are semiconducting, which results in poor electrical conductivity and severely limits their electrocatalytic performance. Heterostructure engineering is one of the effective strategies to overcome these limitations. Constructing intimate heterointerfaces between TMDs and metals, transition metal (oxy)hydroxides (including LDHs), oxides, or conductive carbon can enhance active site modulation, facilitate electron transport, and promote interfacial synergy.

Introducing highly conductive phases that couple coherently with TMDs, or directly inducing transitions to the metallic phase, can markedly accelerate charge transfer and create more electrochemically active sites [138]. Gao et al. grew MoS_2_ in situ on metallic CoSe_2_ surfaces to construct a MoS_2_/CoSe_2_ hybrid interface [139]. Benefiting from the synergistic electrocatalysis between MoS_2_ and CoSe_2_ phases and from a substantially larger population of catalytic sites, the MoS_2_/CoSe_2_ hybrid exhibits high HER activity, with an onset potential of −11 mV and a Tafel slope as low as 36 mV dec^−1^ in acidic media. In MoS_2_–Ni_3_S_2_ heteronanorods, the hierarchical architecture not only exposes highly active heterointerfaces but also facilitates charge transport along the Ni_3_S_2_ nanorods grown on conductive nickel foam, thereby boosting the kinetics and activity toward HER and OER [140]. Zhang et al. further constructed MoS_2_/Ni_3_S_2_ heterostructures through interface engineering [141]. By combining experiments with density functional theory calculations, they revealed that charge redistribution at the interface promoted the chemisorption of hydrogen and oxygen-containing intermediates, thereby accelerating overall electrochemical water splitting.

The work function difference between TMDs and heterostructures drives interfacial charge transfer from the low work function side to the high work function side. This charge redistribution generates an interfacial dipole and a built-in electric field, which in turn optimize the adsorption free energies of intermediates. First-principles studies of MoS_2_/M_2_CO_2_ heterostructures indicate that a moderate work-function difference is most favorable. It yields a balanced interfacial charge transfer and an appropriately strong built-in field that shifts the d-band center toward the Fermi level, thereby enhancing HER activity [142]. Yan et al. fabricated MoSe_2_/MoS_2_ heterostructures, in which interfacial electronic reconstruction shifted the d-band center upward and enhanced the density of states near the Fermi level [143]. This modulation redistributes charge at the active sites and balances the adsorption and desorption of the key H* intermediate, achieving an overpotential of 45 mV at 10 mA cm^−2^ and a Tafel slope of 37.9 mV dec^−1^. Interfacial charge transfer can also act synergistically with water molecules. At the Ni(OH)_2_/MoS_2_ heterointerface reported by Zhang et al., the interfacial charge transfer simultaneously promoted the H_2_O dissociation and the H* adsorption, lowering the barrier of the initial water-splitting step [144]. This hybrid catalyst exhibits excellent HER performance in 1 M KOH, with an onset overpotential of 20 mV, an overpotential of 80 mV at 10 mA cm^−2^, and a Tafel slope of 60 mV dec^−1^.

Heterointerfaces between TMDs and other phases commonly suffer from lattice mismatch, which leaves interfacial atoms under-coordinated and introduces local strain and defects [145]. Such structural distortions in turn generate sulfur vacancies and promote reconstruction and the formation of active sites. By anchoring metallic Co nanodisks onto sulfur-vacancy-rich MoS_2_, Gu et al. exploited the synergy between the metal–MoS_2_ interface and the sulfur vacancies to expose more Mo/S sites for H* adsorption, achieving overpotentials as low as 179 mV in 0.5 M H_2_SO_4_ and 151 mV in 1.0 M KOH, with correspondingly small Tafel slopes (62.5 and 99.8 mV dec^−1^) [146]. Geng et al. activated the interfacial S sites by coupling Ru nanoparticles onto the inert basal plane of MoS_2_ nanosheets [147]. Both DFT calculations and experiments show that the electronic structure of these S sites is modulated, while ΔG_H* results confirm optimized H adsorption on MoS_2_.

#### 4.2.3. TMP-Based Heterointerfaces

In transition metal phosphides (TMPs), the metal sites act as the centers for H* adsorption and desorption, whereas the phosphorus sites act as proton (H^+^) acceptors. They tune the metal–H binding energy toward the thermoneutral optimum. TMPs such as Ni_2_P, CoP, FeP, and MoP have excellent HER activity [148,149]. Under OER conditions, the TMP surface can self-reconstruct into a metal (oxy)hydroxide/phosphate layer, endowing these materials with bifunctional catalytic potential [150]. Nevertheless, at high potentials, TMPs are prone to over-oxidation and dissolution of the active phase, which severely compromises their long-term stability [15]. To overcome these limitations, constructing intimate heterointerfaces between TMPs and metals, transition-metal sulfides, oxides or (oxy)hydroxides/LDHs can markedly enhance water electrolysis performance.

Exploiting the work-function difference between the metallic phase and phosphides to build metal–semiconductor (Mott–Schottky) junctions is a common way to tune the interfacial electronic structure of TMPs. For example, Janus-structured Co/CoP nanoparticles acted as Mott–Schottky electrocatalysts that enable efficient HER and OER over a wide pH range. The work-function difference between metallic Co and semiconducting CoP drives interfacial charge transfer, generating a built-in electric field [151]. When the Co/CoP heterojunction is loaded onto hierarchically ordered porous carbon, the Co/CoP interface not only provides abundant active sites but also optimizes the adsorption free energy of H* and H_2_O through electronic coupling [152]. In 1.0 M KOH, the overpotentials for the hydrogen evolution reaction and the oxygen evolution reaction at 10 mA cm^−2^ are only 120 and 260 mV, respectively. Similarly, Janus-structured Ni/Ni_2_P nanoparticles encapsulated hierarchical N-doped carbon nanofibers have been prepared via a scalable hydroxycarbonate-assisted pyrolysis strategy, enabling high-efficiency OER [153]. Ni/Ni_2_P nanoparticles encapsulated by graphitic carbon have also been demonstrated as efficient bifunctional electrocatalysts for overall water splitting [154].

For phosphide–phosphide interfaces, electronic reconstruction mainly modulates the d-band center and the density of states near the Fermi level [155]. For example, a rationally constructed Cu_3_P/Ni_2_P heterojunction serves as a bifunctional electrocatalyst for both HER and OER [156]. Through heterojunction-induced electronic regulation, it exhibits an OER overpotential of 330 mV at 50 mA cm^−2^ and an HER overpotential of 88.1 mV at 10 mA cm^−2^ in 1 M KOH. P-vacancy-rich Ni_2_P/Cu_3_P interfacial nanosheets deliver excellent overall water-splitting activity, originating from abundant electroactive sites, a built-in electric field at the interface, and P-vacancy-induced electronic-structure reconstruction [157]. A hierarchical Al-CoP/Ni_2_P catalyst was grown in situ on Ni foam (Al-CoP/Ni_2_P@NF) through a combined interface and doping-engineering approach [158]. The heterostructure, Al doping, and hierarchical morphology together optimized the electronic configuration and enlarged the active surface area. The optimized material therefore delivered excellent HER activity in 1.0 M KOH, with overpotentials of only 55 and 72 mV at 10 and 20 mA cm^−2^, and a Tafel slope as low as 55.6 mV dec^−1^. Moreover, both the triphasic Ni_2_P–Fe_2_P–CoP heterointerface and the bifunctional Ni_2_P/NiMoP catalyst optimize the adsorption energetics of HER/OER intermediates through multi-interface electronic coupling, thereby lowering the voltage required for overall water splitting [159,160].

### 4.3. Substrate–Catalyst Layer Interface

In situ growth of catalysts on conductive substrates is an effective strategy for constructing self-supported electrodes, in which the catalyst is anchored to the substrate through chemical bonding or non-covalent interactions. Compared with powder-based electrodes, such binder-free electrodes facilitate electron transport and enhance mechanical robustness. The three-dimensional porous structure of the substrate promotes the high dispersion of the catalyst and the exposure of active sites, while simultaneously providing continuous electron transport channels and efficient pathways for gas–liquid mass transport. Moreover, the interface between the catalytic layer and the conductive substrate can significantly influence electrode performance. On the one hand, this interface regulates the interfacial electron distribution and modulates the adsorption energy of reaction intermediates. On the other hand, it suppresses the migration, detachment, and dissolution of active species, thereby enhancing the long-term stability of the electrode [161]. Herein, carbon-based and metal-based substrates are discussed as two representative cases.

#### 4.3.1. Carbon-Based Substrate

Carbon-based materials with high electrical conductivity, large specific surface area, tunable pore structure, and low cost are widely used as supports for water-splitting catalysts. The performance of a supported catalyst depends not only on the carbon support itself but also on the catalyst–carbon interface, whose electronic structure and charge-transfer behavior are strongly influenced by the support’s surface functional groups, bonding configuration, and structural defects. Because carbon supports differ markedly in these characteristics, the choice of support (e.g., carbon cloth, graphene, or carbon nanotubes) has a pronounced effect on the activity and stability of the resulting electrode.

Pristine carbon cloth is chemically inert and poorly wettable, which hinders the uniform deposition of catalysts and their firm anchoring. Acid oxidation, plasma treatment, and heteroatom doping introduce defects and functional groups that serve as anchoring/nucleation sites for catalysts. Tran et al. treated carbon cloth with a HNO_3_/H_2_SO_4_ mixed acid and observed a marked increase in surface hydroxyl and carboxyl groups together with enhanced hydrophilicity [162]. This treatment enabled a higher and more uniform loading of Au/Co_3_O_4_ (3.36 wt% Co and 1.42 wt% Au) than on untreated carbon cloth, increased the specific surface area to 40.5 m^2^ g^−1^, and reduced the charge-transfer resistance. In 0.5 M H_2_SO_4_, the resulting bifunctional catalyst achieved overall water splitting at only 1.62 V and operated stably for 24 h at 10 mA cm^−2^. Because single-step acid oxidation provides only a limited density of functional groups and thus limited anchoring strength, Mei et al. combined plasma etching with acid treatment to introduce oxygen-containing functional groups while generating additional defect sites [163]. The resulting electrode exhibited a 34.6% lower OER overpotential than pristine carbon cloth, with the Tafel slope decreasing from 97.5 to 55.9 mV dec^−1^.

These anchoring sites not only serve as nucleation centers for the growth of precursor phases (e.g., hydroxides, oxides, or metal–organic frameworks) but also, after the precursors are converted into the active phase by phosphidation or selenization, anchor and stabilize the final catalyst through strong interfacial contact [164]. The resulting in situ-grown self-supported architecture exposes more active sites and provides a direct electron pathway from the conductive carbon cloth to the catalyst, thereby facilitating charge transfer. Song et al. grew cobalt-based MOF arrays on carbon cloth at room temperature and obtained self-supported (Ni,Co)Se_2_ arrays by ion exchange and solvothermal selenization [165]. The electrode achieved a low OER overpotential of 256 mV at 10 mA cm^−2^. Using carbon cloth as the substrate, Liu et al. constructed a composite electrode of FeCoNi layered ternary hydroxides decorated with NiCo_2_O_4_ nanoneedles (FeCo-Ni-LTH/NiCo_2_O_4_/CC) through a facile two-step approach [166]. The electrode delivers an OER onset overpotential of 240 mV and an overpotential of only 302 mV at 50 mA cm^−2^. For HER, it exhibits an onset overpotential of 96 mV and requires 151 mV to reach 20 mA cm^−2^.

Graphene, with its unique sp^2^ carbon framework, ultra-high specific surface area, excellent carrier mobility and good chemical stability, has been widely used to construct self-supported catalysts. Fei et al. reported an atomically dispersed Co–N–C HER catalyst, in which cobalt atoms were anchored on nitrogen-doped graphene and exhibited excellent HER activity in both acidic and alkaline media [167]. The outstanding performance is attributed to the Co–N_x_ coordination structure and the efficient electron-transport pathways provided by highly conductive graphene. When an active phase is loaded on graphene, the graphene π-electron system can hybridize with the metal d orbitals. Tsounis et al. anchored Pt single atoms (mass loading as low as 0.71 μg Pt cm^−2^) on edge-rich, vertically oriented graphene [168]. Angle-resolved X-ray absorption spectroscopy combined with density functional theory indicated that the edge-anchored Pt single atoms were strongly coupled with the π electrons of graphene, increasing the occupancy of the Pt 5d orbitals and shifting the d-band center toward the Fermi level. This strengthened H* binding, significantly improving the alkaline hydrogen evolution performance.

To overcome the weak metal–support interactions, Su et al. introduced transition-metal single atoms on oxygen-doped graphene before loading Ru nanoparticles [169]. The structure significantly strengthened the metal–support interaction and interfacial electronic coupling between Ru nanoparticles and the carbon substrate, facilitated efficient electron transfer from the Ru nanoparticles to the substrate, optimized the hydrogen adsorption energy at the Ru active sites, and ultimately improved the HER performance. Strong metal–support bonds can also be established by introducing heteroatoms or defect sites into graphene. For example, Tang et al. used nitrogen-doped graphene as the framework and utilized the defect sites as nucleation centers [170]. Nanoscale NiFe hydroxides were grown in situ and uniformly distributed within the graphene network. The strong interfacial coupling between the nitrogen sites and the hydroxides not only suppressed the agglomeration and restacking of the active components, but also optimized the electronic structure of the metal centers. The strongly coupled composites exhibit excellent oxygen evolution reaction activity in alkaline media.

#### 4.3.2. Metal-Based Substrate

Three-dimensional metal substrates offer abundant accessible surface and excellent electrical conductivity, making them ideal substrates for the self-supporting electrodes [171]. The three-dimensional porous structure of metallic foams not only enlarges the electrochemical active area but also promotes gas-bubble detachment. Taking nickel foam as an example, active phases can be grown directly in situ by hydrothermal growth, electrodeposition, or vapor-phase oxidation–reduction, thereby achieving strong interfacial coupling, high activity, and mechanical stability [172]. The metal foam can also act as a metal source and self-sacrificial template for the active phase. Kong et al. prepared nanoporous Ni foam through an oxidation treatment of commercial Ni foam [173]. In situ growth of NiFe-LDH on the porous structure exposed abundant nucleation sites and enhanced H_2_O adsorption, achieving an OER overpotential of only 270 mV at 100 mA cm^−2^ and stable operation for over 2200 h. Li et al. proposed a polyoxometalate-mediated electroplating–activation strategy to construct a self-supported molybdenum nickel oxide electrode for the OER [174]. The active layer anchors firmly on the Ni foam current collector with sufficient surface exposure and high structural stability, thus delivering high catalytic efficiency in alkaline media. Bu et al. prepared Co_9_S_8_–Ni_3_S_2_ heterostructures via an MOF-derived sulfidation route, in which the heterointerfaces facilitate interfacial electron transfer and increase the electrochemical surface area [175]. It achieved a low OER overpotential of 233 mV. Ma et al. constructed Fe_4.4_Ni_17.6_Se_16_/NiSe self-supporting electrodes by selenizing porous FeNi alloy foam, with overpotentials of only 242 and 282 mV at current densities of 100 and 500 mA cm^−2^, respectively [176].

Compared with nickel foam, copper foam has lower cost, higher conductivity, and a unique electronic structure. It is another widely used three-dimensional self-supporting substrate, with applications ranging from simple hydrogen/oxygen evolution to CO_2_ reduction, nitrate reduction, and other processes [177]. Cu foam-derived electrodes can be transformed in situ through oxidation, sulfidation, phosphidation, or alloying, thereby creating abundant interfacial active sites while preserving the three-dimensional framework [178]. Ma et al. roughened the surface of three-dimensional Cu foam via sulfidation, thereby generating copper sulfide active phases in situ [179]. The resulting bifunctional self-supporting electrode was used for overall water splitting. Its 3D porous structure enlarged the electrochemically active surface area and accelerated gas-bubble release, further enhancing the catalytic efficiency. Huang et al. synthesized a bifunctional electrocatalyst via a facile hydrothermal–phosphidation route [180]. The catalyst consisted of iron-doped CoP heterostructures self-assembled on copper foam. Benefiting from the synergistic Co–Fe effect, the favorable metal–phosphorus bonding that tunes the electronic structure, and a large electrochemically active surface area, CoFeP/CF achieved outstanding performance, requiring only 277.9 mV (OER) and 152.6 mV (HER) to reach 50 mA cm^−2^ in alkaline electrolyte.

## 5. Perspective

Interface engineering has proven effective for enhancing the electrocatalytic performance of water splitting, enabling interface-engineered electrocatalysts to attain activity comparable to or even exceeding that of noble-metal catalysts such as Pt/C. Nevertheless, the rational design of high-performance electrocatalysts through interfacial engineering still faces considerable challenges:

1. Resolving dynamic interfacial structures under operating conditions.

Currently, in situ techniques for probing interfacial structure remain limited. In situ X-ray diffraction mainly tracks long-range lattice evolution (e.g., phase transitions, lattice strain, and grain size). In contrast, in situ Raman spectroscopy mainly provides local vibrational information (e.g., surface species, defects, and reconstructed phases). Used individually, these techniques generally cannot fully resolve interfacial atomic configurations, charge redistribution, and active-site electronic states. Future studies should combine complementary operando methods, including X-ray absorption spectroscopy, Raman spectroscopy, and, where experimentally feasible, near-ambient-pressure XPS and electrochemical liquid-cell electron microscopy [181]. Measurements should be correlated with electrochemical responses under comparable conditions, while accounting for differences in probing depth, temporal resolution, and possible measurement-induced perturbations. This approach would help distinguish beneficial reconstruction from irreversible degradation and establish more reliable interface–activity relationships.

2. Developing realistic computational models and predictive descriptors.

Theoretical modeling of interfacial systems is limited by two main challenges. On the one hand, the large supercells required for interface models render DFT calculations prohibitively expensive; multiscale simulations and machine-learning potentials offer a way to reduce this cost. On the other hand, for interfaces where charge transfer and lattice strain coexist, a single descriptor such as the d-band center cannot accurately capture the structure–activity relationship. Therefore, interface-specific descriptors are needed to predict intermediate adsorption energies and reaction barriers. These should be validated against structural and electronic-state data from in situ characterization, giving deeper insight into interface structure–performance relationships.

3. Targeted design of coupled interfaces.

A single interface has limited modulating capacity and can hardly optimize charge transfer, intermediate adsorption, and structural stability simultaneously. Multi-interface architectures can circumvent this limitation, yet the contributions of the coupled interfaces are difficult to disentangle, so their design remains largely empirical. To move beyond trial-and-error toward targeted design, future efforts should clarify interface formation mechanisms and quantify the role of each interface. This could be achieved through hierarchical interface construction and multi-site synergy, together with precise fabrication techniques such as atomic layer deposition (ALD).

4. From laboratory to industrial application.

A central challenge is retaining the interfacial composition, coordination environment, and structural integrity established in laboratory studies when catalyst production and electrode area are increased. Non-uniform catalyst deposition, as well as changes introduced during electrode processing, can cause the actual interface to deviate from the intended design. Furthermore, under high current densities, vigorous gas evolution, mass transport limitations, and catalyst degradation can further diminish the performance advantages observed in laboratory testing.

Future work should prioritize scalable synthesis and coating methods, strengthen catalyst–support adhesion, and optimize electrode porosity and wettability to maintain reactant transport and gas release. For example, NiMoO_4_-based electrodes with an epitaxial catalytic layer have been operated in an industrial alkaline electrolyzer at 450 mA cm^−2^ for 1400 h, providing a representative example of translating interface design into practical devices [182]. As an enabling tool spanning synthesis, characterization, and evaluation, AI can help explore complex interfacial compositions and optimize preparation conditions. Therefore, a dataset linking interfacial structures, synthesis parameters, catalytic activity, durability, and device performance should be constructed. Active learning can then combine model prediction, material synthesis, structural characterization, and electrochemical testing, accelerating catalyst discovery and optimization.

We surveyed interface-engineered electrocatalysts for water splitting. Their key performance metrics are summarized in Table 3, with emphasis on catalysts that show industrial promise.

## 6. Conclusions

This review focuses on the regulatory mechanisms of interface effects in electrochemical water splitting, systematically summarizing the principles of interface engineering and the latest research progress in enhancing water splitting performance. Centered on three typical interfaces—core–shell structures, transition-metal-based heterostructures, and substrate–catalyst interfaces—this article summarizes their structural characteristics and interface regulation strategies, and highlights how interface structures can synergistically regulate the electronic structure of active sites and the adsorption/desorption behavior of reaction intermediates through lattice strain, interfacial charge redistribution, and orbital hybridization effects, thereby simultaneously improving the activity and stability of both the HER and OER.

In the future, the controllable construction of interface structures will provide a pathway to low-cost, highly stable hydrogen production via water electrolysis. This would also facilitate the integration of water electrolysis with renewable energy sources such as solar and wind power.

## Figures and Tables

**Table 1:** Core–shell structured HER electrocatalysts.

Catalyst	Electrolyte	Overpotential	Tafel Slope	Long-Term Stability	Ref.
CoP@Ni	1.0 M KOH	71 mV @ 10 mA cm^−2^	66 mV dec^−1^	——	[97]
Fe-rich MEA@MEO	1.0 M KOH	89.9 mV @ 10 mA cm^−2^	67.6 mV dec^−1^	−1000 mA cm^−2^, 50 h	[98]
PtCoNiMoRh@Rh	Acid/neutral/alkaline	9.1/24.9/17.1 mV @ 10 mA cm^−2^	31.4 mV dec^−1^	——	[99]
MoP/MoS_2_/NF	1.0 M KOH	90 mV @ 10 mA cm^−2^	99 mV dec^−1^	——	[100]
Cu@Cu-Ni-Co	1.0 M KOH	125 mV @ 10 mA cm^−2^	79.1 mV dec^−1^	270 mA cm^−2^,120 h	[101]
NiS@MoS_2_/NC	1.0 M KOH	189 mV @ 10 mA cm^−2^	56.7 mV dec^−1^	——	[102]
L1_0_-PtCo@Co	0.5 M H_2_SO_4_	13 mV @ 10 mA cm^−2^	27.2 mV dec^−1^	150 mA cm^−2^, 450 h	[103]
VS_2_/Ti_3_C_2_	0.5 M H_2_SO_4_	59 mV @ 10 mA cm^−2^	38.42 mV dec^−1^	150 mA cm^−2^, 48 h	[104]
Double-shell hollow carbon@Ru nanoreactors	1.0 M KOH	73.5 mV @ 10 mA cm^−2^	83.7 mV dec^−1^	100 mA cm^−2^, 24 h	[89]
Pt_tet_@Ni(OH)_2_	1.0 M KOH	——	27 mV dec^−1^	——	[91]
Ni-MoS_2_@ NiS_2_@Ni_3_S_2_	1.0 M KOH	69.4 mV @ 10 mA cm^−2^	64.1 mV dec^−1^	500 mA cm^−2^, 168 h	[105]
HMCs@Ru	Acid00/neutral/alkaline	64.3/78.1/2.8 mV @ 10 mA cm^−2^	32 mV dec^−1^ (in alkaline)	——	[106]

The symbol “——” in the table indicates that the corresponding content is not mentioned in the paper.

**Table 2:** Core–shell structured OER electrocatalysts.

Catalyst	Electrolyte	Overpotential	Tafel Slope	Long-Term Stability	Ref.
CoP-12@NiMoO_4_/NF	1.0 M KOH	165 mV @ 10 mA cm^−2^	45 mV dec^−1^	——	[116]
NiFeMo2C@C	0.5 M KOH	292 mV @ 10 mA cm^−2^	65.3 mV dec^−1^	10 mA cm^−2^, 24 h	[117]
Cu@Cu(OH)_2_ nanorods/MOF	1.0 M KOH	310 mV @ 10 mA cm^−2^	167.54 mV dec^−1^	——	[118]
NiP@NiPOx/Fe(OH)_3_	1.0 M KOH	149.1 mV @ 10 mA cm^−2^	67.6 mV dec^−1^	500 mA cm^−2^, 500 h	[119]
Ce-doped NiO@MoO_2_	1.0 M KOH	163 mV @ 10 mA cm^−2^	18.7 mV dec^−1^	1000 mA cm^−2^, 200 h	[120]
CoOOH@Co_3_S_4_	1.0 M KOH	292 mV @ 100 mA cm^−2^	132 mV dec^−1^	100 mA cm^−2^, 72 h	[121]
CoNi-LDH @COAC/NF	1.0 M KOH	253 mV @ 10 mA cm^−2^	42.26 mV dec^−1^	10 mA cm^−2^, 100 h	[122]
Co@Fe-P	1.0 M KOH	280 mV @ 10 mA cm^−2^	41.9 mV dec^−1^	100 mA cm^−2^, 60 h	[123]
Fe-Ni_2_B@C/NF	1.0 M KOH	208 mV @ 10 mA cm^−2^	49 mV dec^−1^	2000 mA cm^−2^, 1000 h	[124]
RuO_2_@TaO_x_	acidic	163 mV @ 10 mA cm^−2^	62.6 mV dec^−1^	——	[125]
FeOOH@Ni_3_N	1.0 M KOH	209 mV @ 10 mA cm^−2^	38.9 mV dec^−1^	500 mA cm^−2^, 100 h	[112]
Co_3_Fe_7_/Fe_3_O_4_	1.0 M KOH	279 mV @ 10 mA cm^−2^	42.88 mV dec^−1^	——	[126]
Cu@Ag@ Co(OH)_2_/CF	1.0 M KOH	324 mV @ 100 mA cm^−2^	76 mV dec^−1^	100 mA cm^−2^, 40 h	[127]

The symbol “——” in the table indicates that the corresponding content is not mentioned in the paper.

**Table 3:** Representative experimental advances in interface engineering.

Catalyst	Preparation Strategy	Reaction and Test Conditions	Performance	Long-Term Stability	Ref.
e-NiMoO_4_	Hydrothermal processing followed by electrochemical synthesis	1 M KOH; industrial-type alkaline electrolyzer: 30 wt% KOH, 50 °C, 2 MPa (HER)	η_10_ = 32 mV U = 2.14 V @ 0.40 A cm^−2^	1400 h @ 0.45 A cm^−2^	[182]
Ru@Cu-TM	Self-supported arrays grown by electrochemical deposition	1 M NaOH (HER)	η = 85 mV @ 200 mA cm^−2^; U = 1.69 V @ 1 A cm^−2^; 2.00 V @ 3.6 A cm^−2^	>300 h @ 1 A cm^−2^	[183]
CN@NiCoS	Hydrothermal precursor growth + gas-phase sulfidation with in situ carbon coating	1 M KOH; 1 M KOH + natural seawater (HER)	η_10_ = 4.6 mV, η_1000_ = 236 mV; seawater: η_1000_ = 281 mV	>1000 h @ 100 mA cm^−2^; alkaline seawater: >200 h @ 1 A cm^−2^	[184]
NiFe LDH/Fe_2_(MoO_4_)_3_	One-step water bath process by immersing nickel foam in a Ni/Fe/Mo-containing precursor solution	1 M KOH, 25 °C; AEMWE: 3 M KOH, 60 °C (OER)	η = 316 mV @ 2 A cm^−2^; U = 2.00 V @ 2 A cm^−2^; 1.70 V @ 1 A cm^−2^	3000 h @ 2 A cm^−2^, 4.5 μV h^−1^; AEMWE: 100 h @ 1 A cm^−2^	[185]
W_1_–NiFeOOH	EquiformerV2 equivariant graph neural network; ML-potential screening of 3976 configurations	1 M KOH; AEMWE 80 °C, long-term durability: 60 °C (OER)	η_10_ = 178 mV; 80 °C: U = 1.56 V @ 1 A cm^−2^; 2.00 V @ 13.1 A cm^−2^	>500 h @ 1 A cm^−2^, 60 °C (AEMWE)	[186]

## Data Availability

No new data were created or analyzed in this study. Data sharing is not applicable to this article.
